# Removal of Azithromycin from Aqueous Solution Using UV- Light Alone and UV Plus Persulfate (UV/Na2S2O8) Processes

**Published:** 2018

**Authors:** Mehraban Sadeghi, Ramezan Sadeghi, Bahareh Ghasemi, Gashtasb Mardani, Ali Ahmadi

**Affiliations:** a *Department of Environmental Health of Engineering, Faculty of Health, Shahrekord University of Medical Sciences, Shahrekord, Iran.*; b *Department of Statistic and Epidemiology, Faculty of Health, Shahrekord University of Medical Sciences, Shahrekord, Iran.*

**Keywords:** Azithromycin, Ultraviolet, Sulfate radicals, Persulfate

## Abstract

Azithromycin is among the broad-spectrum antibiotics that is widely available in various environmental systems and could have destructive effects on the ecosystem and human health due to its bacterial resistance. In this study, removal of azithromycin from wastewater using an advanced oxidation process of ultraviolet light with and without persulfate was investigated and effective parameters for the management of each of the processes were evaluated. The effect of different parameters including the concentration of Azithromycin antibiotic at levels 5, 15, 45 mgL^-1^; the concentration of persulfate at levels 1, 2, 4 mmol; pH at levels 5, 7, 9, contact time in 30, 60, 90 minute range of azithromycin removal was investigated. Ultraviolet light at a wavelength of 254 nanometers was used to irradiate the reactor. The results showed that azithromycin removal was significantly lower in the presence of ultraviolet radiation alone 58% with the removal efficiency than the case that ultraviolet radiation was used with sodium persulfate 98%. The best azithromycin removal conditions were obtained at the removal efficiency with the initial concentration of antibiotic 5 mgL^-1^, the concentration of persulfate 1mmol, and the contact time 30 min. and pH = 7. The rate of decrease in the concentration of residual azithromycin is increasing with increasing sodium persulfate concentration and decreasing the initial azithromycin concentration. This research can help to apply the integrated use of advanced oxidation processes to idealize decomposition-resistant compounds removal processes and to better understand the parameters affecting the removal.

## Introduction

Population growth and social and economic development have caused serious damage to the environment in the past 50 years (1). Most medications, including antibiotics, are highly stable in the environment, so their residual presence in the environment, especially in water resources, has become an important problem (2, 3). Antibiotics enter the environment through the household and hospital wastewater, sewage of veterinary clinics and agricultural runoff into sewage collection systems and the environment (4). An average of 70% of the consumed antibiotics enters the sewage without any changes (5). Urban and hospital wastewater treatment plants are not sufficiently effective in eliminating these compounds, therefore the antibiotics can enter the water sources (6). 

The expired or unused drug compounds may enter the landfill site. If the right technology is not used in the designing, developing or equipping these sites, their leakage to the surface and groundwater and contaminating the drinking water sources will be inevitable. Biological solids from the sediments in pounds of wastewater treatment plants contain high concentration of these compounds (7, 8). Moreover, there are evidences of accumulation of antibiotics in the environment (9). However, many drug contaminants such as antibiotics and their metabolites can cause complications, such as endocrine dysfunction and microorganism resistance (10, 11). Therefore, today in many review articles the subjects of occurrence and fate of drugs in the environment as well as the effects on humans and other organisms in the environment are emphasized (12). Azithromycin, erythromycin, clarithromycin are among the most important antibiotics in the macrolide group and are widely used in medicine and veterinary clinics (13, 14, 15).

 Azithromycin is the result of a change in the erythromycin structure. This compound has different chemical and antimicrobial properties compared to erythromycin, for example, it is more stable in the acidic environments and it is better diffused in tissues. A large amount of this drug is not metabolized in the body and is largely eliminated by the bile (16, 17). Various methods such as adsorbents, chemical compounds such as chlorine and photochemical oxidation are used to remove antibiotics entered into the aquatic environments (18-20). Advanced oxidation processes have been widely used as effective methods for degradation and removal of hazardous and persistent pollutants in many aquatic environments. During these processes free radicals, such as hydroxyl and sulfate radicals, are produced, leading to the decomposition and reduction of toxicity of organic compounds in various environments such as wastewater (21, 22). Sulfate radicals produced in advanced oxidation processes have recently been considered by many researchers and are discussed as an alternative to reduce organic pollutants because they enjoy a high efficiency in removing pollutants that cannot be removed by conventional methods (16). Other advantages of sulfate radicals include high stability, selectivity power, and high electron volts (2.6 ev), moderate costs, and being eco-friendly (22-24). Persulfates are widely used as a precursor of sulfate radicals due to their specific advantages, including relatively low costs, high water solubility, easy storage and pre-activation transportation. Several methods, such as ultraviolet radiation, temperature, intermediate metals, and ultrasonic waves can activate persulfates and produce sulfate radicals (25). Today, advanced oxidation processes are considered in decomposing various pollutants such as medications during which by employing ultrasound, ultraviolet radiation, nanoparticles, and heat. The persulfates are used to produce sulfate radical (Reduction potential equal to 2.6 V) (16, 21, 25-30).

The aim of this study was the comparative removal of azithromycin from sewage using two ultraviolet and ultraviolet/sodium peroxide processes based on the formation of hydroxyl and sulfate radicals. Parameters such as initial concentration of antibiotic, initial persulfate concentration, reaction medium, and reaction time were used as effective factors on system conductance and after obtaining the optimal conditions for the best removal efficiency experiments were evaluated on real wastewater as well. 

By obtaining the COD, the removal efficiency of other organic compounds in the sewage was also measured by the combined process of ultraviolet and persulfate radiation. Also, nitrate, phosphate, and bicarbonate anions removal efficiency was also calculated from this process.

## Experimental

*Chemicals*

**Figure 1:**
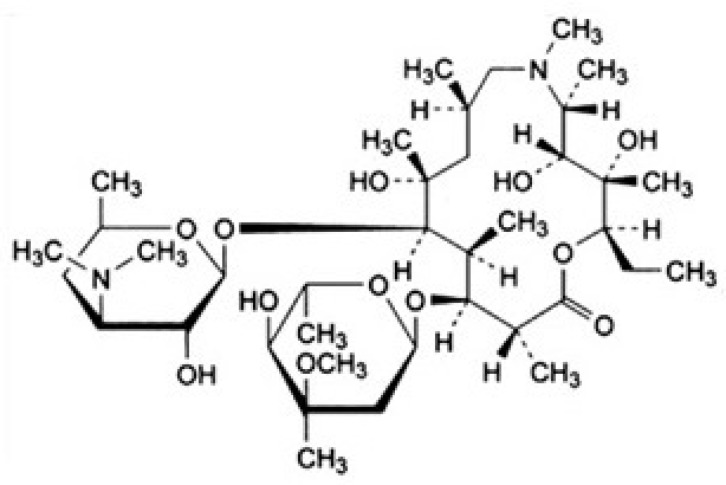
Structure of azithromycin (31)

Azithromycin (C_38_H_72_N_2_O_12_) 98%, Analytical grade, CAS Number: 117772-70-0 was purchased from the Farma Pharmaceuticals Company Tehran, Iran. The molecular structure of azithromycin is shown in Figure 1. Sodium persulfate (Na_2_S_2_O_8_, 99%), CAS Number: 7775-27-1, of the Sigma Aldrich Company HPLC grade methanol (CH_3_OH; ≥ 99.9%), CAS Number: 67-56-1, acetonitrile (CH_3_CN; ≥ 99.5%), CAS Number: 75-05-8, sodium hydroxide (NaOH; ≥ 99.8%), CAS Number: 1310-73-2, and sulfuric acid (H_2_SO_4_; ≥ 98%), CAS Number: 7664-93-9, were purchased from Merck-Germany. Standard Stocks Solution of 1000 mgml^-1^ of antibiotic was prepared by solving 100 mg of azithromycin antibiotic in 100 mL of methanol. Standard Stocks Solution of 50 mmol was prepared by solving 11.9g Sodium persulfate in one liter of deionized water with a resistivity less than 5 Ω. (C18, 6 ml, 500 mg), and also solid phase extraction (SPE) tubes were purchased from INOPAK, Korea. Low pressure mercury lamp (manufactured in OSRAM- Italy) was purchased with a protective quartz coating (6 w, wavelength of 254 nm).

*Experimental procedure*

The experiments were carried out in a calibrated glass reactor (250 mL volume). The low pressure mercury lamp (6w, wavelength of 254 nm) with a quartz protective tube was submerged in the middle of the reactor’s central cylinder. The effect of ambient light was prevented by wrapping this set in an aluminum foil. The continuous mixing of the specimen was performed by the magnetic stirrer. After preparation of the reactor, an appropriate volume of antibiotic stocks and sodium persulfate solutions were added. Using the sulfuric acid and sodium hydroxide 0.1 N, the pH of the sample was adjusted within the desired range. By turning the mercury lamp on and during the contact time 30, 60, 90 minutes; the entire contents of the reactor were passed through the cartridge. In order to ensure the decomposability, all tests were repeated twice and the standard deviation was considered to be less than 5%.

In this study also for investigating the effectiveness of advanced oxidation (ultraviolet/persulfate and ultraviolet radiation) on the actual wastewater of hospitals a wastewater specimen was collected from hospital in Shahr-e-Kord and analyzed after the transfer to the laboratory. The specimen was first filtered through 0.45 µm filter in the laboratory. The steps of SPE tube preparation, extraction and analysis of specimens were carried out according to the analytical method described in the next section. The concentration of antibiotic studied in the hospital wastewater 5.04 mgL^-1 ^was reported. To compare the two methods of ultraviolet and ultraviolet/persulfate, all conditions were considered the same.

**Table 1:** Characteristics of the real samples collected from hospital wastewater before oxidation and after that

**Characteristics of the real wastewater**	**Concentration (mgL** **-1** **)**	
	**Before oxidation**	**After oxidation**
Chemical Oxygen Demand	415	138
Bicarbonate	300.08	0
Nitrate	78.97	44.22
Phosphate	2.82	1.21

**Figure 2:**
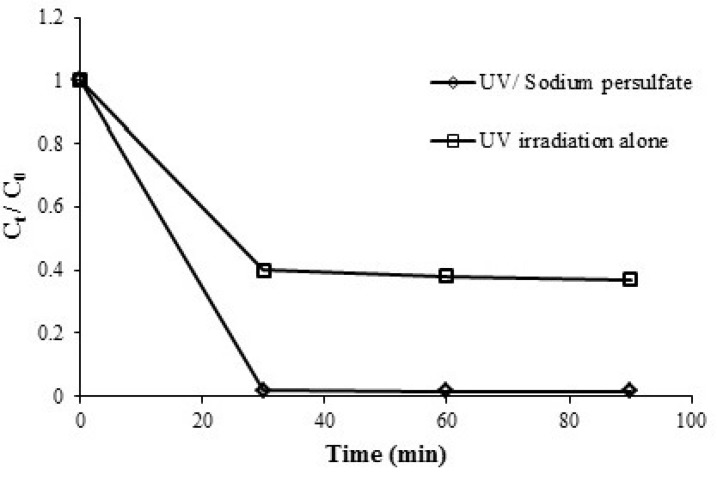
Removal of azithromycin by ultra violet/persulfate and ultra violet alone processes, azithromycin initial concentration= 5 mgL-1, persulfate concentration= 1mmol, and pH = 7

**Figure 3:**
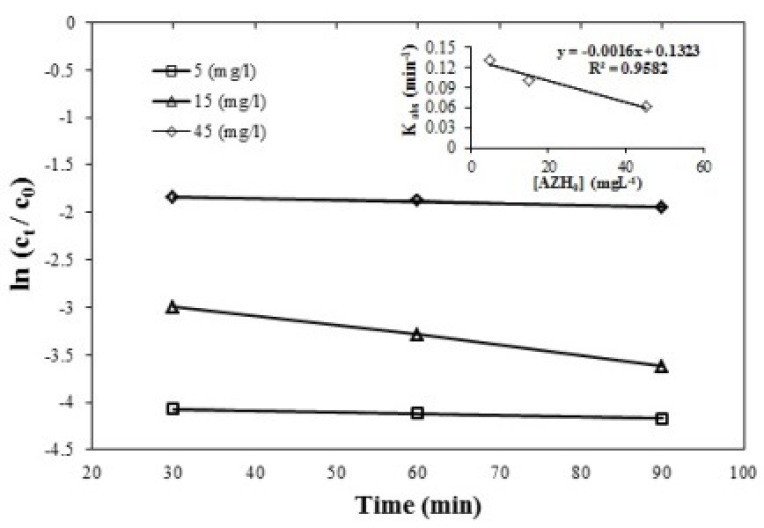
**. **Effect of initial concentration of azithromycin on the removal efficiency, azithromycin initial concentration = 5, 15, and 45 mgL-1, persulfate initial concentration=1 mmol, and pH=7, (R2 = 0.9582)

**Figure 4:**
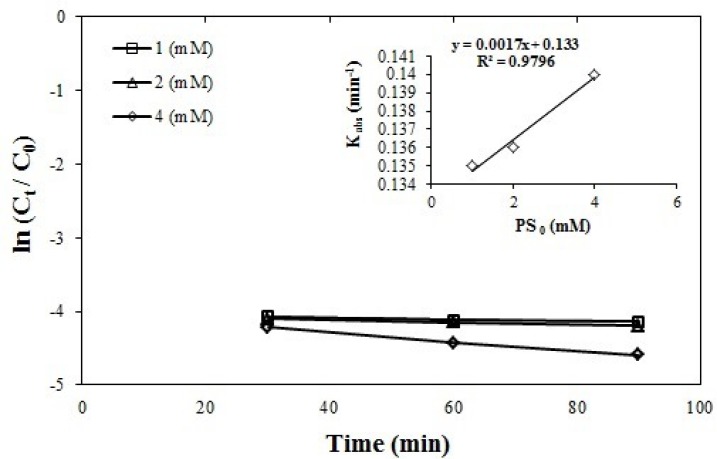
Effect of initial concentration of persulfate on azithromycin removal efficiency, azithromycin initial concentration = 5 mgL-1, persulfate initial concentration = 1–4 mmol, and pH = 7, (R2 = 0.9796)

**Figure 5:**
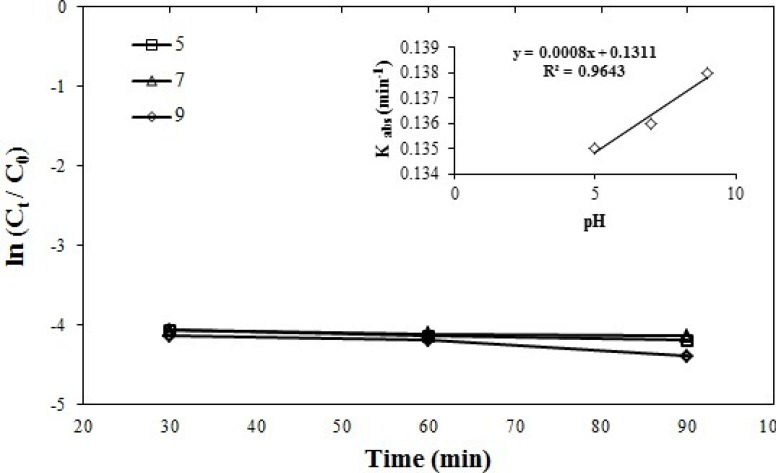
Effect of pH on azithromycin removal efficiency, azithromycin initial concentration = 5 mgL-1, persulfate initial concentration = 1 mmol, and pH = 5, 7, and 9, (R2 = 0.9643)

**Figure 6:**
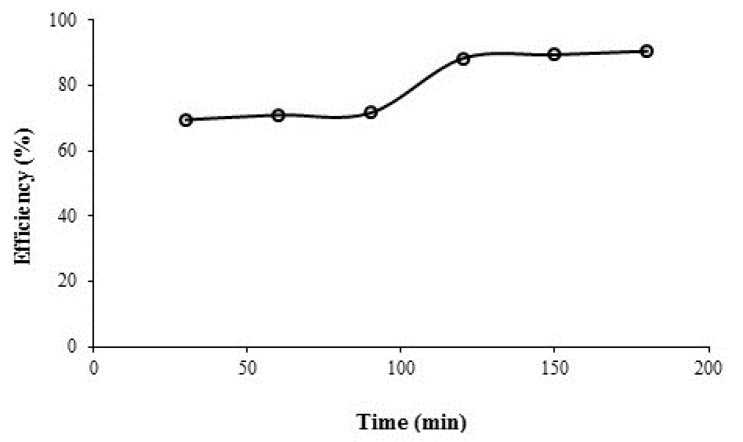
Azithromycin removal efficiency vs. oxidation time in a real wastewater collected from hospital, azithromycin initial concentration = 5.04 mgL-1, persulfate initial concentration = 1 mmol, and pH = 7

**Figure 7:**
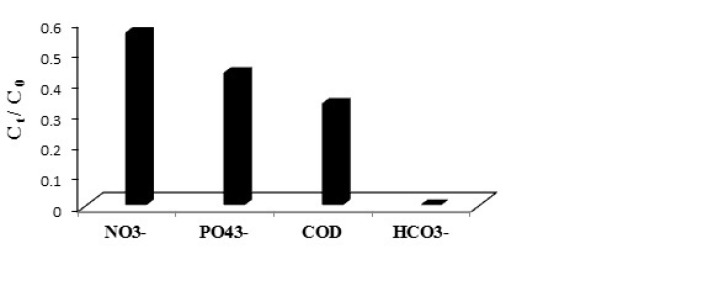
Ratios of the rate constants in the presence and the absence of a specific anion (k/k0) at different anion concentrations, azithromycin initial concentration=5.04 mgL-1, persulfate initial concentration = 1 mmol, pH = 7, and contact time = 30 min

*Analytical methods*

High Performance Liquid Chromatography equipped with fluorescence detector (HPLC FLD, Agilent series 1200) and column C18 (particle size 5 μm, internal diameter 4.6 mm and length 150 mm) was used in analytical experiments. A mixture of methanol and 0.1% formic acid in the volumetric ratio of (90:10) was used as the mobile phase in a uniform flow of 0.3 ml/min (31). The injected sample size was 20 μl and Isocratic elution was used. In order to separate, purify, and concentrate of contaminants from samples, many methods including SPE were used (32-35). For this purpose, the SPE tube containing the appropriate adsorbent (silica gel) was selected. For the preparation of SPE tubes, 5 ml of methanol and 5 mL of deionized water with less than 5 Ω resistance was passed through them. 200 mL of the filtered sample was added to the flow rate of 1 mL/min to the SPE tube and after the removal of the sample and rinsing the tube, 2 ml of acetonitrile was used for washing and separating the remaining antibiotic from the adsorbent (36, 37). During all these stages the manifold and a vacuum pump were used. 

The extracted specimen was collected in a Microtube 2 mL for HPLC injection. 20 μL of the extracted specimens were injected into an HPLC device through a 20 μL loop. Using the equation and calibration diagram, the amount of residual azithromycin was calculated after the advanced oxidation process and using the following equation 1, the amount of azithromycin removal efficiency was measured. The other analysis such as COD, bicarbonate, nitrate, and phosphate were performed based on the protocol in standard methods for the examination of water and wastewater; (38).

Efficiency (%) = $\frac{Ct-C0}{\mathrm{C0}}$ ×100                                                          Equ. 1

The obtained data were entered into the SPSS software and according to the type of data distribution ANOVA and Bonferroni tests were used. The significance level was considered as pv < 0.05. 

## Results and Discussion

*Comparison of Azithromycin removal by direct photolysis UV alone and UV/persulfate*

Azithromycin removal in any of the processes (i.e. UV alone and UV/sodium persulfate) is shown in Figure 2. The removal rate for the initial concentration of 5 mgL^-1^ obtained under the same conditions in terms of pH = 7, PS = 1 mmol, time = 30 min. 58% and 98% for UV alone and UV/sodium persulfate, respectively. Decrease the amount of Azithromycin followed first order kinetics. In general, the reaction rate constant obtained from the equation 2, was increased when ultraviolet radiation was used alone 0.03 min^-1^ compared to the case that the ultraviolet light was used with sodium persulfate 0.13 min^-1^. Increasing the efficiency in UV/sodium persulfate process could be due to the production of sulfate radicals due to the simultaneous use of sodium persulfate and ultraviolet radiation along with the presence of hydroxyl radicals. As a result, the degradation and removal of azithromycin in optimal conditions is increased from 58% (UV alone) to 99% (UV/persulfate). 

The study Yu-Qiong Gao *et al*. on sulfamethazin showed that among the ultraviolet, persulfate, ultraviolet and persulfate and ultraviolet and hydrogen peroxide removal methods the highest removal efficiency was associated with 96.5% the ultraviolet and persulfate process (30). Similar results were observed by Yang Deng *et al*. on fluorophenicol showed that compared to the UV and UV/persulfate methods, the UV/persulfate method removed 98.4% fluorophenic over an hour (39). The study Moussa Mahdi Ahmed, Serge Chiron on carbamazepine showed that the highest removal efficiency was associated with the UV/persulfate method (40).

-K_abs_. t= Ln $\frac{\left[\mathrm{AZH}\right]t}{\left[\mathrm{AZH}\right]0}$                                                           Equ.2

where;

[AZH]_t_: Azithromycin concentration at time

[AZH]_0_: Initial Azithromycin Concentration

K_abs_: First order reactions constant, the minus sign in front of the k_abs_ term is because the concentration of AZH is decreasing over time.

*Effect of initial azithromycin concentration on the removal efficiency*

Based on the studies, the reaction’s constant was reduced according to the equation 

K=) -0.0016 [AZH_0_] + 0.1323) from 0.13 min^-1^ at a concentration of 5 mgL^-1^ to 0.061 min^-1^ at a concentration of 45 mgL^-1^ of the azithromycin antibiotic. Azithromycin removal efficiency was compared at three levels of antibiotic concentration 5, 15, 45 mgL^-1^ by one-way ANOVA. According to the Figure 3 there was a significant difference between the concentrations 5, 45 mgL^-1^ of azithromycin; the p-value was 0.00 (first type error). With the increase in antibiotic levels, the decrease in antibiotic elimination efficiency is observed. The possible justification of these changes can be because the available sulfate radicals are insufficient to degrade the high concentrations of azithromycin antibiotic (41). 

Kordatou Michael *et al*. on the study of erythromycin removal efficacy with UV/persulfate process, it was concluded that the removal efficiency was reduced by increasing the antibiotic concentration (42). Similar results were observed by the study Shengnan Su *et al.* of amoxicillin that with the increase in amoxicillin concentration, the removal efficiency is reduced (22).

*Effect of initial sodium persulfate concentration on the removal efficiency*

The azithromycin removal efficiency was compared at three levels of sodium peroxide concentration (1, 2, 4 mmol) using one-way ANOVA and no statistically significant difference was observed between the three levels of concentration. 

The reactions constant was increase according to the equation K = 0.0017 [PS_0_] + 0.133 for 0.135 min^-1^ at a concentration of 1 mmol to 0.14 min^-1^ at a concentration of 4 mmol of the sodium persulfate. These changes are presented in the Figure 4 which is first-order linear. The statistical distribution of the obtained data was normal and was obtained by performing one-way ANOVA. Considering that there is no significant difference between the selected concentrations, from the economic point of view, the concentration of 1 mmol of sodium persulfate was chosen as the optimum concentration because this concentration provides higher efficiency with low difference with other concentrations 90%. 

Yu-qiong, Gao *et al*. in their study on the removal of sulfamethazine and fluorophenicol from water by persulfate and ultraviolet concluded that the concentration of 1 mmol of sodium persulfate was the optimal concentration (30, 39).

*Effect of pH on azithromycin removal efficiency*

The reactions constant was obtained in K = -0.0008 pH + 0.1310, 0.135 min^-1^ at a pH = 5, 0.136 min^-1^ at pH = 7, 0.138 min^-1^ at pH = 9. This suggests that UV/persulfate process has similar efficiency in a wide spectrum pH (acidic, neutral and alkaline) which is one of the advantages of the UV/persulfate method. As it can be observed in the Figure 5. since there is no significant difference between the selected levels and all three diagrams are tangent to each other, the best and most economical level in three levels 5, 7, 9 is neutral (pH = 7) due to the lack of use of acid or alkali to adjust with the reactive environment. Irina Appalled *et al.* in a study conducted on levoflacxin by ferrous and persulfate, they also concluded that they have a better efficiency in near-neutral pH state (43). Similarly, Minghua Nie *et al*. the study on chloramphenicol antibiotics showed that the highest removal efficiency is obtained in the neutral pH state (44).

*Effect of contact time on azithromycin removal efficiency*

According to the Figure 3-5. Showing azithromycin removal efficiency versus time for different conditions of pH and initial concentration of azithromycin and persulfate, after exceeding 30 min, more than 90% of the removal process has been completed. Although this process improves over 90 minutes, the impact of and difference between these times are not so significant (PV > 0.05) to cause cost and time. 

Xiaoli Zou *et al*. in a study on sulfadiazine removal efficacy found that at the time of 30-60 minutes the removal efficiencies are close to each other (45). In a study conducted by Shengnan Su *et al*. on amoxicillin removal using persulfate radicals, in the first 30 minutes, the process of eliminating antibiotics was 50% (22).

*Azithromycin removal from real sample*

Figure 6 presents the changes in azithromycin removal between contact times 30-180 min in a real sample and optimal conditions (i.e. pH = 7, contact time = 30 min., and persulfate concentration =1 mmol). The amount of removal is approximately constant at 70% and between 30-90 min and 10% there is an incremental process of 90-120 min in removal efficiency and again for 120 min the removal efficiency continues without any substantial change. The results showed that the UV/persulfate process can remove 69.44% of azithromycin in the wastewater in optimal conditions. As noted above, the highest removal efficiency occurred in the synthetic sample in 30 min. However, the removal efficiency in the real sample the same period is reduced by 30% due to the presence of other organic compounds in the real wastewater. 

In order to obtain higher azithromycin removal efficacy in real conditions the period of the reaction stay in optimal condition was increased to 60, 90, 120, 150, and 180 min. As it can be observed in Figure 6 efficiency has increased to 70.92%, 71.54%, 83.22%, 85.35%, and 89.46%, respectively. With the increase in contact time, the most significant difference was observed at 120 min., which may be explained by the removal of other organic compounds by sulfate radicals within the time interval 30-90 min., and then the removal of the stable compound of the azithromycin antibiotic occurred which was maximized within a period of 2 h. The curve shows that although with increasing the time we can see that the increase in the removal efficiency was resulted, but this change in the efficiency is very gradual and there isn’t a significant difference between the removal percent at 120 min and those at 180 min. It is observed that there is a significant difference between the efficiency of antibiotic removal in the real wastewater samples and those in the synthetic wastewater samples in the same conditions in terms of the oxidation process. Perhaps the existence of some other anions and organic compounds in the real samples is the reason for this difference. Table 1 shows the effect of UV/persulfate process on the COD, phosphate, nitrate and bicarbonate removal in the real samples of hospital wastewater.

The nitrate in the wastewater is converted to the nitrate radicals by UV/persulfate process, and decreases with the efficiency 44%. Due to the creation of an internal filter, it reduces the amount of sulfate radicals and, as a result, reduces azithromycin removal efficiency compared to the time when the synthetic sewage without nitrates was analyzed (32). The conditions for the phosphate present in the sewage are in the same way, with the difference that more phosphate is affected by the superficial process, which is equal to 57%. The carbonates and bicarbonates in the sewage can also compete with antibiotics over the sulfate radicals. So that 100% of these anions are won and removed during the competition. The removal efficiency of organic compounds in the wastewater was obtained 66.74% (COD decrease from 415 to 138 mgL^-1^), which indicates oxidant demand of the organic compounds and decreasing the removal efficiency of azithromycin in the raw wastewater (Figure 7). The competition between the mentioned above compounds with azithromycin content can be shown as follows.

HCO_3_^-^> COD> PO_4_^3-^> NO_3_^-^

## Conclusions

The use of UV/persulfate process on a laboratory scale was a safe method to remove azithromycin. In this study, the effect of azithromycin concentration, persulfate concentration, pH and contact time on azithromycin removal efficiency was investigated. In the conducted experiments the azithromycin antibiotic concentration of 5 mgL^-1^, sodium peroxide concentration of 1 mmol, pH = 7 and the contact time of 30 min were obtained as optimal values. The ultraviolet radiation process removes 60.21% and UV/ sodium persulfate process removes 98.30% of azithromycin antibiotic in synthetic wastewater under the optimal conditions. This study proved that the idealization of the removal process can affect the efficiency of the system so that the removal efficiency in optimal conditions of the real wastewater was obtained as 70% and with the increase of the contact time to 3 h it approached to 90%. The study demonstrated the use of advanced UV/persulfate oxidation is a good way to remove azithromycin and similar antibiotics in the hospital and pharmaceutical plant’s wastewater. We propose more studies to apply this oxidation method and to determine the operation parameters for similar antibiotics in pilot and full scale in the future. 
